# The risk associated with ultra-processed food intake on depressive symptoms and mental health in older adults: a target trial emulation

**DOI:** 10.1186/s12916-025-04002-4

**Published:** 2025-03-24

**Authors:** Belayneh Mengist, Mojtaba Lotfaliany, Julie A. Pasco, Bruno Agustini, Michael Berk, Malcolm Forbes, Melissa M. Lane, Suzanne G. Orchard, Joanne Ryan, Alice J. Owen, Robyn L. Woods, John J. McNeil, Mohammadreza Mohebbi

**Affiliations:** 1 Deakin University, the Institute for Mental and Physical Health and Clinical Translation (IMPACT), School of Medicine, Geelong, VIC Australia; 2 College of Medicine and Health Sciences, Debre Markos University, Debre Markos, Ethiopia; 3 School of Public Health and Preventive Medicine, Monash University, Melbourne, VIC Australia; 4 Department of Medicine–Western Health, The University of Melbourne, St Albans, Victoria, Australia; 5 Biostatistics Unit, Faculty of Health, Deakin University, Geelong, VIC Australia

**Keywords:** Depression, Mental health, Target trial emulation, Ultra-processed food

## Abstract

**Background:**

Longitudinal cohort studies across the lifespan suggest an association between ultra-processed food (UPF) and depression. However, the effect of UPF on depression and mental health in older adults has not been determined. Therefore, this study investigated the effect of UPF on depressive symptoms and mental health in community-dwelling older adults.

**Methods:**

A pragmatic target trial was designed and emulated using the ASPirin in Reducing Events in the Elderly longitudinal data. Participants were community-dwelling older adults (≥ 70 years) in Australia. We specified and emulated the protocol of a two-arm randomised pragmatic clinical trial using the level of UPF consumption as the intervention. Greater than or equal to 4 servings of UPF per day was considered the intervention, with less than 4 servings per day the control. Dietary consumption was assessed using a mail-based diet screening questionnaire, and the level of food processing was classified based on the NOVA classification. The study outcomes were depressive symptoms, defined as a score of ≥ 8 on the Center for Epidemiological Studies Depression 10-item scale, and general mental health, defined by the mental component summary score of the Short Form-12. We applied inverse probability treatment weighting to balance confounders. Marginal structural models were employed to estimate the population-level average effect of intervention using generalised estimated equations.

**Results:**

A total of 11,192 participants (3415 intervention and 7777 control) were eligible for the emulation. High UPF consumption at time zero was associated with an increased risk of depressive symptoms at follow-ups (RR: 1.10; CI: 1.04–1.18). The finding was consistent with sensitivity analyses; after excluding participants on antidepressants at time zero, the risk of depressive symptoms in the intervention group was increased by 11% compared to the control (RR: 1.11; 95% CI: (1.04–1.20)). Consumption of UPF adversely affected the mental component quality of life (*β*: − 0.40; CI: − 0.65 to − 0.15).

**Conclusions:**

A higher level of UPF consumption was associated with a higher risk of depressive symptoms and adversely affected mental health among older adults.

**Supplementary Information:**

The online version contains supplementary material available at 10.1186/s12916-025-04002-4.

## Background

Given the rapid rise of the older population, mental disorders are a major public health concern for this population. According to a recent World Health Organisation (WHO) report, approximately 14% of older adults live with mental disorders, contributing to 10.6% of the total years lived with disability worldwide [1]. Although depression affects individuals at any stage of life, late-life depression is a common mental health condition, often under-recognised and undertreated [1], associated with several adverse health outcomes [2]. Its pathophysiology and risk pathways remain incompletely understood [3].

Diet is a modifiable factor for health. Consumption of a low-quality diet contributes to several health and health-related conditions in older adults including functional disability [4], sarcopenia [5, 6], frailty [7], depression [8], cognitive decline [9, 10], and chronic diseases [11, 12]. The mechanism by which diet is associated with these health outcomes is complex and multifaceted, but diet impacts many pathways implicated in mood [13].

According to the NOVA classification, a widely used food grouping system noting the nature, extent, and purpose of industrial processing, food can be classified into four groups: (i) unprocessed or minimally processed food; (ii) processed culinary ingredients; (iii) processed food; and (iv) ultra-processed foods (UPF) [14]. UPF are manufactured foods generally characterised by high energy and poor nutritional profiles, containing a mix of additives to give long shelf life and to make them attractive and palatable, which include confectionary sweets, sweetened beverages and packaged/ready-meals [14, 15].

UPF is typically nutritionally unbalanced in terms of nutrient composition with high added sugar, fat and trans-fat constructed from extracted, refined, fractionated and low-cost ingredients [16, 17]. Moreover, processing techniques such as artificialisation (use of colourants, flavours, artificial sweeteners, emulsifiers, cosmetic additives, and synthetic food products) as well as a transformation of food attributes to include non-nutritious products [17].

The dietary pattern of the global population is changing with an increase in UPF consumption. In high-income countries, including the United States of America (USA) and Australia, over half of the total energy intake is through UPF [17, 18]. A study among older adults in the Netherlands reported that 37% of total energy intake was from UPF [19].

Extant literature demonstrates that UPF is associated with a range of adverse health outcomes, including dementia [15], mental health disorders [20], cardiovascular diseases, cancer, type 2 diabetes, frailty, chronic inflammation and all-cause mortality [18, 21, 22].

An umbrella review on UPF and adverse health outcomes indicated that UPF is associated with adverse mental health outcomes such as sleep-related problems, anxiety and incident depression [23]. The review classified the level of evidence for UPF consumption on depression as low. Similarly, a systematic review and meta-analysis showed that a higher UPF consumption was associated with increased odds of common mental disorders (depressive and anxiety symptoms) [24]. Moreover, in a recently published cohort study among British adults (*n* = 4554, 1183 females, mean age of 61 ± 5.9 years), a higher intake of UPF was associated with a 34% higher likelihood of recurrent depressive symptoms [25].

Although these studies have found an association between UPF and depressive symptoms, the effect of UPF on the development of depressive symptoms among older adults has not been thoroughly investigated. Given the prevalence of late-life depression and a globally ageing population, assessing the effect of diet on mental health outcomes in older adults considering potential effect modifiers and confounders, including socio-demographics, comorbidities, and biomarkers, is crucial to enhance healthy ageing. Conducting randomised controlled trials (RCTs) to investigate the effect of diet on health outcomes is not straightforward due to the feasibility of large-scale RCTs, challenges in dealing with information bias, expectation bias due to the knowledge of which study participants are receiving intervention or control [26, 27] and ethical issues [28]. Causal interpretation from observational studies is challenging due to the lack of randomised treatment assignments [29]. Target trial emulation is a framework to apply principles of randomised trials to prospective longitudinal observational studies and uses inverse probability of treatment weighting (IPTW) to control for measured confounders through balancing potential confounders of exposed and unexposed groups [30].

This study aimed to employ a target trial framework to address the methodological limitations of observational studies and to investigate the effect of UPF on depressive symptoms and mental health quality of life in older adults using longitudinal data from the ASPrin in Reducing Events in the Elderly study (ASPREE).

## Methods

We designed an emulated two-arm randomised pragmatic clinical trial using the target trial framework to assess the effect of UPF on depressive symptoms in older adults.

### Data sources

This study was a secondary analysis of the ASPREE trial. ASPREE was a double-blind randomised placebo-controlled trial of daily 100 mg enteric-coated aspirin in 19,114 community-dwelling older adults aged 70 + years from Australia and 65 + years from the USA. Participants were males and females willing and able to provide informed consent recruited between 2010 and 2014, and the clinical trial was completed in June 2017. Participants were recruited from primary care in Australia and predominantly through academic and clinical trial centres in the USA. Participants were free from overt cardiovascular disease, dementia, and independence-limiting physical disability at baseline. The design, recruitment, eligibility criteria and baseline characteristics of participants in the ASPREE study have been published in detail [31–33].

The ASPREE Longitudinal Study of Older Persons (ALSOP) is a sub-study of the ASPREE trial among Australian participants (*n* = 16,703) that started in early 2012, where 12,578 participants provided the year 3 medical and lifestyle data including the diet screen survey. The details of the ALSOP study have been previously published [34]. The follow-up of participants after the trial phase continued through the ASPREE-eXTension (ASPREE-XT) study and investigates the long-lasting effects of aspirin and a broad range of factors that contribute to healthy ageing [35].

**Fig. 1:**
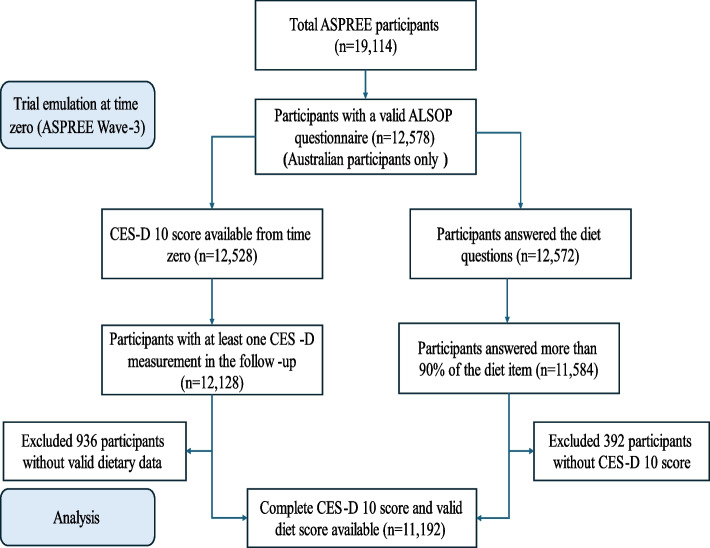
Flow diagram showing ASPREE and ALSOP participants used in the analysis

The longitudinal dataset contains socio-demographic characteristics, social and physical health indicators, anthropometrics, medical diagnoses, medications, laboratory tests and dietary information, among other data [35]. We used ASPREE participants with valid ALSOP diet data and incorporated extended follow-ups beyond the ASPREE trial period from the ASPREE-XT starting from wave 3 of the ASPREE trial (time zero of this study) to ASPREE-XT 04 (wave 11) (Fig. 1).

### Study design and approach

A target trial emulation was applied based on the ASPREE longitudinal data. The study protocol for a hypothetical randomised control trial was framed by rigorously defining eligibility criteria, assignment procedures (intervention and control arms), follow-up, outcome ascertainment and analysis plan. The components of the target trial design are summarised in (Table 1).

**Table 1:** Summary of a target trial protocol and its emulations to estimate the effect of ultra-processed food on depressive symptoms in older adults using ASPREE data

Protocol component	Target trial	Target trial emulation
Eligibility criteria	**Inclusion:** Community-dwelling older adults (> 70 years) and no upper age limit **Exclusion:** Follow-up commenced when all eligibility criteria were met	Same as the target trial Participants with no valid dietary score were excluded In sensitivity analysis, participants taking antidepressants and/or antipsychotics at time zero and participants with clinically relevant depressive symptoms at time zero were excluded
Intervention strategy	Strategy 1: consume a high level of UPF (intervention) at time zero of the trial Strategy 2: consume a low level of UPF (control) at time zero	Same as the target trial UPF consumption was calculated in servings per day and categorised as high consumption (≥ 4 servings/day) and low consumption (< 4 servings/day) We assumed that the diet habits of older people were stable so that participants maintained the allocation groups
Intervention assignment	Participants were randomly assigned to the intervention at time zero and aware of the group to which they were assigned (randomisation but no blinding)	We assumed random assignment of high or low UPF intake by employing inverse probability weights to balance predefined confounders, a-priori selected from the ASPREE data domains
Outcomes	Onset of depressive symptoms Mental component quality of life	Same as the target trial We defined depressive symptoms as the presence of clinically relevant depressive symptoms measured by a total score of ≥ 8 on the CES-D 10 scale during the follow-up period Mental component quality of life was assessed using the SF-12
Follow-up	We followed participants after randomisation (intervention assignment) until the first episode of depressive symptoms, lost follow-up, or end of follow up whichever occurs first	Same as the target trial Time zero was defined at wave 3 of follow-up, when the diet survey was collected, and ended at the last available follow-up (APREE-XT 04)
Causal contrast	Intention-to-treat	Intention-to-treat effect (effect of intervention as specified in the protocol)
Statistical analysis	Intention-to-treat analysis	Intention-to-treat analysis was applied. We used logistic regression to calculate IPTWs and the marginal structural model to estimate the population average effect of intervention through generalised estimated equations

*IPTW* Inverse probability treatment weights

### Eligibility criteria

We included community-dwelling older adults of the ASPREE participants who completed the dietary habits food screening survey as part of the ALSOP sub-study. To be eligible for the target trial, participants had to be assessed for depression endpoints in the ASPREE study and had a valid food screening survey. In sensitivity analyses, we considered the following as exclusion criteria: participants who had depressive symptoms at time zero and participants taking antidepressants and/or antipsychotics at time zero. In this study, wave-3 of the ASPREE trial was considered as a baseline (labelled as time zero in the manuscript) as the dietary assessment was conducted at year-3 of the trial period.

### Intervention strategies

We compared two dichotomised intervention strategies. The first strategy involved consuming a high level of UPF (intervention) at the initiation of the target trial (i.e. time zero), and the second strategy involved consuming a low level of UPF over the same period (control).

### Dietary assessment

Dietary information was assessed using a mail-based diet screening questionnaire. The questionnaire was used to assess the dietary intake of participants that contains food and drink items categorised into food groups such as meat, fish and eggs, snack and convenience foods, dairy (including milk and milk alternatives)), bread, grains and cereals, fruit and vegetables, drinks (soft drinks, cordial, supplements drinks, etc.) and other nutrients (salt, fats and oils, water, and discretionary foods) based on the expert knowledge of dietary patterns of older adults. Consumption frequency was recorded considering the last 12-month diet in the form of scales ranging from never to every day/several times a day.

The level of food processing was determined according to the NOVA food classification system [14]. Foods and drinks of the screener tool were classified into four groups (unprocessed/minimally processed, culinary ingredients, processed food, and ultra-processed food). Then, 21 food and drink items were identified as UPF (NOVA group 4), which includes processed meats (e.g. bacon, ham, corned beef or salami), sausages, potato chips or similar, sweet biscuits/cakes, dark chocolate, milk chocolate, lollies or other sweets, hamburgers/pizza or 'fast' food, meat pies or sausage rolls, ice cream, frozen yoghurt or other dairy desserts, breakfast cereal/oats, crackers/savoury biscuits, mass-produced packaged bread, soy or other non-dairy milk, malt drinks (e.g. Milo or Horlicks), cordial, soft drink (e.g. regular Coke), hot chocolate, diet soft drink (e.g. Diet Coke) and supplement drinks (e.g. Ensure or Sustagen) (Additional file 1: Table S1).

Data from the diet screen were converted to a daily equivalent frequency (DEF) for each food using the reference guide adapted from the Victorian Cancer Council food frequency questionnaire user guide for dietary questionnaires for epidemiological studies. Daily UPF consumption was calculated as serving per day by converting consumption of UPF frequencies from the diet screen response to a daily frequency to make all foods in similar units as; 0 for never or almost never, 0.053 for one to two times a month, 0.21 for one to two times a week, 0.64 for three to six times a week, and 1 for every day of the week. For drinks, we used 0 for never or almost never, 0.14 for once per week or less, 0.57 for several times a week, 1.5 for one to two times a day, and 3 for three or more times a day [36]. Serving per day for each participant was computed by summing the serving score of each UPF item. Then UPF consumption was categorised as high consumption (≥ 4 servings/day) and low consumption (< 4 servings/day) according to a recent study conducted to investigate the association between UPF consumption and risk of depression [37]. Sex-specific quartiles were also computed to stratify UPF consumption (Additional file 1: Table S2).

In addition, an estimated portion size (the amount of food in grams that a person consumed at a particular eating time) of UPF consumption was considered. Age- and sex-specific median portion sizes in grams were estimated (for older adults; 71 + years) by multiplying the daily consumption frequencies for each item by its specified portions [38] based on the Australian National Nutrition and Physical Activity Survey report [39]. The estimated portion size for UPF consumption (grams/day) was calculated by summing up portions of each UPF item. The weighted proportion of UPF was calculated as UPF consumed divided by the total amount of food consumed in grams per day using similar methods used by previous studies [40, 41]. Quartiles of portion size as a daily energy percentage contribution of UPF were used for the analysis.

### Trial interventions

Eligible participants of the target trial were assumed a random allocation to one of the two dietary intervention strategies: (i) exposed (intervention) group—participants who were consuming high levels of UPF (≥ 4 servings per day) were taken as the intervention arm and (ii) control group—participants who consumed low levels of UPF (< 4 servings per day) were taken as the control arm. We considered the 12-month dietary data a stable dietary pattern [42] and performed an intention-to-treat analysis, assuming participants maintained the assigned intervention strategy for the duration of the study.

### Outcomes and follow-up

The primary outcome of the target trial was the risk of depressive symptoms. Participants were followed from time zero of the emulated trial (i.e. wave-3 of the ASPREE study) to 2022 (up to a maximum of wave-11 of ASPREE-XT 04). We followed up with the participants until the onset of depressive symptoms, the last available follow-up, lost to follow-up or 2022 (the time on which wave-11 of ASPREE-XT 04 data were collected) whichever occurred first.

The depression outcome was defined using the Center for Epidemiologic Studies-Depression 10-item (CES-D 10) scale [33, 43]. The CES-D 10 is a self-administered questionnaire of 10 items on depressive symptoms, which range from 0 to 30, with higher scores indicating more severe depressive symptoms. The scale was validated for older adults previously [43, 44]. Depressive symptom was defined as the presence of clinically relevant depressive symptoms measured by a total score of ≥ 8 on the CES-D 10 scale [45, 46]. The CES-D 10 was administered annually, including the ASPREE-XT 04 visit (2022).

The secondary outcome of interest was mental health quality of life which was measured using the 12-item Short Form Health Survey (SF-12). The SF-12 health survey is a 12-item summary score assessing physical and mental health quality [47]. The Mental Component Score (MCS) score was generated by combining medical outcome short-form items using the US general population-derived item weights [48]. The SF-12 MCS ranges from 0 to 100, with higher scores indicating better mental health functions and being a valid measure of depression [49]. The SF-12 was administered annually through the ASPREE-XT 04 visit.

### Covariates

Potential confounders include age, sex, ethnicity (race), years of education, smoking status, alcohol use, living situation (living alone or living with family/in a residential home), self-reported physical activity, social support [50], Body mass index (BMI) in kg/m^2^, waist circumference, CES-D score at time zero, global cognitive function (Modified Mini-Mental State examination (3MS) scored out of 100), comorbidities (hypertension, diabetes mellitus, dyslipidaemia, chronic kidney disease (CKD), gastro-oesophageal reflux disease (GORD), pulmonary disease, gout, cancer and Parkinson’s disease), multimorbidity (coexistence of two or more chronic health conditions), metabolic syndrome (based on the Adult Treatment Panel III (ATP III) diagnostic criteria [51], polypharmacy (use of five or more prescription medications) and biomarkers such as lipid profiles and fasting blood glucose. We used causal-directed acyclic graphs (DAGs) to a priori identify the confounders (Additional file 2: Fig. S1).

### Statistical analysis

The primary analysis compared the risk of depressive symptoms among participants who consumed high (intervention arm) and low (control arm) UPF. We used descriptive analyses to present participants’ characteristics by frequencies with percentages for categorical variables and mean with standard deviation (SD) for continuous variables.

The probability of receiving treatment was predicted using multivariable logistic regression considering UPF consumption as an outcome variable conditional on prespecified covariates, and potential interaction terms were checked.

We applied the IPTW approach to create synthetic populations in which the treatment group is independent of measured baseline confounders. Employing IPTW resulted in an intervention and control arms with a similar probability of receiving a high or low level of UPF, and as such, the target population closely mimics the characteristics of a population in a pragmatic randomised trial [52, 53]. We used stabilised weights (SW) to mitigate the impact of selection bias and maintain the robustness of the estimation [53].

To maintain the exchangeability assumption (i.e. participants in the intervention and control group have the same potential outcomes on average), the distribution of confounders across the trial arms was compared after employing IPTW using standardised mean difference (SMD) [54]. An SMD less than 0.1 indicates a covariate balance between the intervention and control group [55]. Weight truncation was applied at the 1st and 99th percentiles (1%) to prevent the effect of extreme weights [52].

For the primary outcome, inverse probability weights were included in the marginal structural model (MSM) by employing the generalised estimated equations with a log link, CES-D 10 ≥ 8 as the dichotomised outcome, and intervention group as the nominal independent variable using the first-order autoregressive correlation structure. Risk ratio (RR) with its 95% confidence interval was used to estimate the effect of UPF on depressive symptoms.

For the secondary outcome, MSM was fitted using generalised estimated equations assuming a Gaussian distribution with an identity link function and repeated (follow-up) MCS SF-12 scores as a continuous outcome variable using the first-order autoregressive working correlation structure. The estimated between-group mean difference with 95% confidence interval was reported. A two-sided *p*-value less than 0.05 was used to determine statistical significance.

To evaluate the extent to which unmeasured confounders could affect the findings, E-values were reported [56, 57].

Sub-group analyses were conducted using sex, level of education, BMI category, presence of multimorbidity (coexistence of two or more chronic health conditions), metabolic syndrome and aspirin use as these are known factors associated with depressive symptoms. We performed sensitivity analyses using quartiles of UPF servings and portion size, using CES-D 10 cutoff ≥ 10 as the outcome, excluding participants with clinically relevant depressive symptoms or those taking antidepressants or/and antipsychotics at time zero. The main analysis was replicated using the ASPREE trial duration only (participants followed until 2017, using four waves) to be consistent with previous studies that reported negligible variability in diet patterns in four years [37, 58].

Statistical analyses were conducted using R statistical software (*ipw* and *geepack* packages), version 4.3.3 (R Foundation for Statistical Computing, Vienna, Austria).

### Missing data

Participants with missing data in the intervention (no diet data at time zero) and outcomes (those with no records at time zero and at least one of the follow-ups) variables were excluded from the analyses. To handle missing data in covariates, we performed imputation using the Predictive Mean Matching (PMM) approach in the *mice* package in R.

## Results

### Participants characteristics

Among 12,597 participants enrolled in the ALSOP sub-study, 11,192 were eligible for this study.

The mean UPF consumption (servings per day) was 3.4 ± 1.5. UPF use was higher among males than among females (3.7 ± 1.6 for men and 3.2 ± 1.4 for females). Three thousand four hundred fifteen participants (30.5%) had high UPF consumption (≥ 4 servings/day) and were classified as the intervention arm.

At the baseline of the trial, the mean (standard deviation) age of participants was 74.9 (4.07) years and 53.7% were females. In our study, at time zero, most participants (*n* = 8490, 90.1%) had good social support, and 8322 (74.8%) were alcohol users at time zero. Most of the participants had two or more medical comorbidities (*n* = 7523, 81.5%; 81.9% from intervention and 81.4% from the control arm). At time zero, 4665 (41.7%) of participants reported polypharmacy use (Table 2).

**Table 2:** Baseline characteristics of ASPREE study participants included in the target trial emulation stratified by intervention strategies before and after IPTW

**Covariates**		**Overall cohort** (frequency/%)	**Unweighted**			**Weighted**		
			Intervention (frequency/%)	Control (frequency/%)	SMD	Intervention (frequency/%)	Control (frequency/%)	SMD
*N*		11,192	3415	7777		11,183	11,195	
Age (years): mean ± SD		74.9 ± 4.07	75.3 ± 4.25	74.68 ± 3.97	0.160	74.94 ± 4.02	74.90 ± 4.12	0.010
Sex: female		6017 (53.7)	1466/42.9	4551/58.5	0.316	5988/53.6	6016/53.7	0.003
Education status: > 12 years		4682 (41.8)	1423/41.7	3259/41.9	0.005	4649/41.6	4675/41.8	0.004
Racial: White		11,059/98.8	3386/99.2	7673/98.7	0.052	11,054/98.8	11,055/98.8	0.001
Alcohol: yes		8322/74.8	2435/71.7	5887/76.1	0.102	8321/74.4	8363/74.7	0.007
Smoking: yes		252/2.3	48/1.4	204/2.6	0.087	252/2.2	249/2.2	0.002
Income: low		8855/92.8	2729/93.4	6126/92.5	0.032	10,352/92.6	10,367/92.6	0.001
Social support: good		8490/90.1	2581/89.7	5909/90.2	0.018	10,033/89.7	10,054/89.8	0.003
Living status: living at home with family		7525/67.6	2443/71.9	5082/65.7	0.134	7560/67.5	7561/67.6	0.001
Physical activity: moderate to high		5953 /61.8	1838/62.4	4115/61.6	0.017	6811/60.9	6874/61.4	0.010
BMI, kg/m^2^: mean ± SD		27.6 ± 4.5	27.3 ± 4.3	27.8 ± 4.6	0.096	27.67 ± 4.7	27.63 ± 4.5	0.008
Waist circumference, cm: mean ± SD		96.5 ± 12.7	96.9 ± 12.5	96.3 ± 12.8	0.052	96.53 ± 12.55	96.63 ± 13.02	0.008
Total cholesterol, mg/dl: mean ± SD		197.7 ± 38.7	194.9 ± 37.7	198.9 ± 39.1	0.103	197.59 ± 37.96	197.55 ± 39.02	0.001
HDL, mg/dL: mean ± SD		61.6 ± 17.8	59.0 ± 17.0	62.8 ± 18.0	0.215	61.66 ± 18.65	61.55 ± 17.69	0.006
Triglyceride, mg/dl: mean ± SD		116.9 ± 55.5	117.4 ± 57.1	116.7 ± 54.7	0.013	117.71 ± 57.13	117.24 ± 55.36	0.008
Blood glucose, mg/dL: mean ± SD		99.1 ± 17.5	98.7 ± 16.6	99.3 ± 17.8	0.031	99.54 ± 18.40	99.52 ± 18.22	0.002
Metabolic syndrome: yes		5000/54.8	1487/52.5	3513/55.8	0.066	6,148/54.9	6164/55.1	0.004
3MS score: mean ± SD		94.4 ± 4.6	94.1 ± 4.9	94.6 ± 4.5	0.118	94.42 ± 4.7	94.4 ± 4.6	0.004
CES-D score at time zero: mean ± SD		4.1 ± 3.9	4.2 ± 3.9	4.06 ± 3.9	0.048	4.12 ± 3.85	4.12 ± 3.94	< 0.001
Multimorbidity: yes		7523/81.5	2337/81.9	5186/81.4	0.015	9145/81.8	9178/82.0	0.005
Comorbidities	Hypertension: yes	8734/78.9	2633/78.0	6101/79.4	0.033	8819/78.9	8828/78.9	< 0.001
	Diabetes: yes	949/9.7	258/8.6	691/10.2	0.055	1099/9.8	1083/9.7	0.005
	Cancer: yes	2555/22.9	845/24.8	1710/22.0	0.065	2525/22.6	2556/22.8	0.006
	CKD: yes	2331/23.2	747/24.0	1584/22.8	0.030	2568/23.0	2564/22.9	0.002
	GORD: yes	3627/32.4	1187/34.8	2440/31.4	0.072	3634/32.5	3642/32.5	0.001
	Dyslipidaemia: yes	6487/66.8	1969/65.6	4518/67.3	0.037	7446/66.6	7434/66.4	0.004
	Parkinsonism: yes	152/1.4	50/1.5	102/1.3	0.013	142/1.3	150/1.3	0.006
	Pulmonary disease: yes	1629/14.6	528/15.5	1101/14.2	0.037	1652/14.8	1632/14.6	0.006
	Gout: yes	541/4.8	181/5.3	360/4.6	0.031	550/4.9	544/4.9	0.002
Polypharmacy: yes		4665/41.7	1399/41.0	3266/42.0	0.025	4703/42.1	4678/41.8	0.005

*CKD* chronic kidney disease, *GORD* gastro-oesophageal reflux disease, *HDL* high-density lipoprotein, *SMD* standardised mean difference, *3MS* Modified Mini-Mental State examination score, *BMI* body mass index in kilogramme/metre square

Physical activity: usual intensity of physical activity in a typical week; low (never, rare or light) and moderate (moderate to vigorous)

Social support: based on the Lubben Social Network Scale (LSNS-6, ≥ 12 out of 30 points was considered as good)

Multimorbidity: coexistence of two or more chronic health conditions (hypertension, diabetes mellitus, dyslipidaemia, chronic kidney disease, gastroesophageal reflux disease, respiratory condition, osteoarthritis, gout, and Parkinson’s disease)

Metabolic syndrome: based on the Adult Treatment Panel III (ATP III) diagnostic criteria as a composite measure of 5 variables (waist circumference, blood pressure, triglyceride, HDL, cholesterol level and blood glucose)

Hypertension: SBP ≥ 140 mmHg or DBP ≥ 90 mmHg or on treatment for high blood pressure

Diabetes: self-report of diabetes or fasting glucose ≥ 126 mg/dL or on treatment for diabetes

Cancer: the diagnosis of any cancer during the study period or a history of a cancer diagnosis by self-report

CKD: an estimated glomerular filtration rate of less than 60 mL/min/1.73 m^2^

Diabetes: self-report or the use of any drug use for the treatment of diabetes, including insulin, or a fasting blood glucose level of greater than or equal to 7 mmol/L

Medication use: was self-report and drugs were classified according to the Anatomical Therapeutic Chemical (ATC) system, including N05A, N06A, C10 and A10

Polypharmacy: taking ≥ 5 prescription medications daily

IPTW was based on propensity score adjusted for age, sex, education status, racial white, alcohol, smoking, income, social support, living status, physical activity, BMI, metabolic syndrome, cognition status, multimorbidity, and polypharmacy after multiple imputation

Weighted SMD was presented for those covariates included in the propensity score model considering multicollinearity

**Fig. 2:**
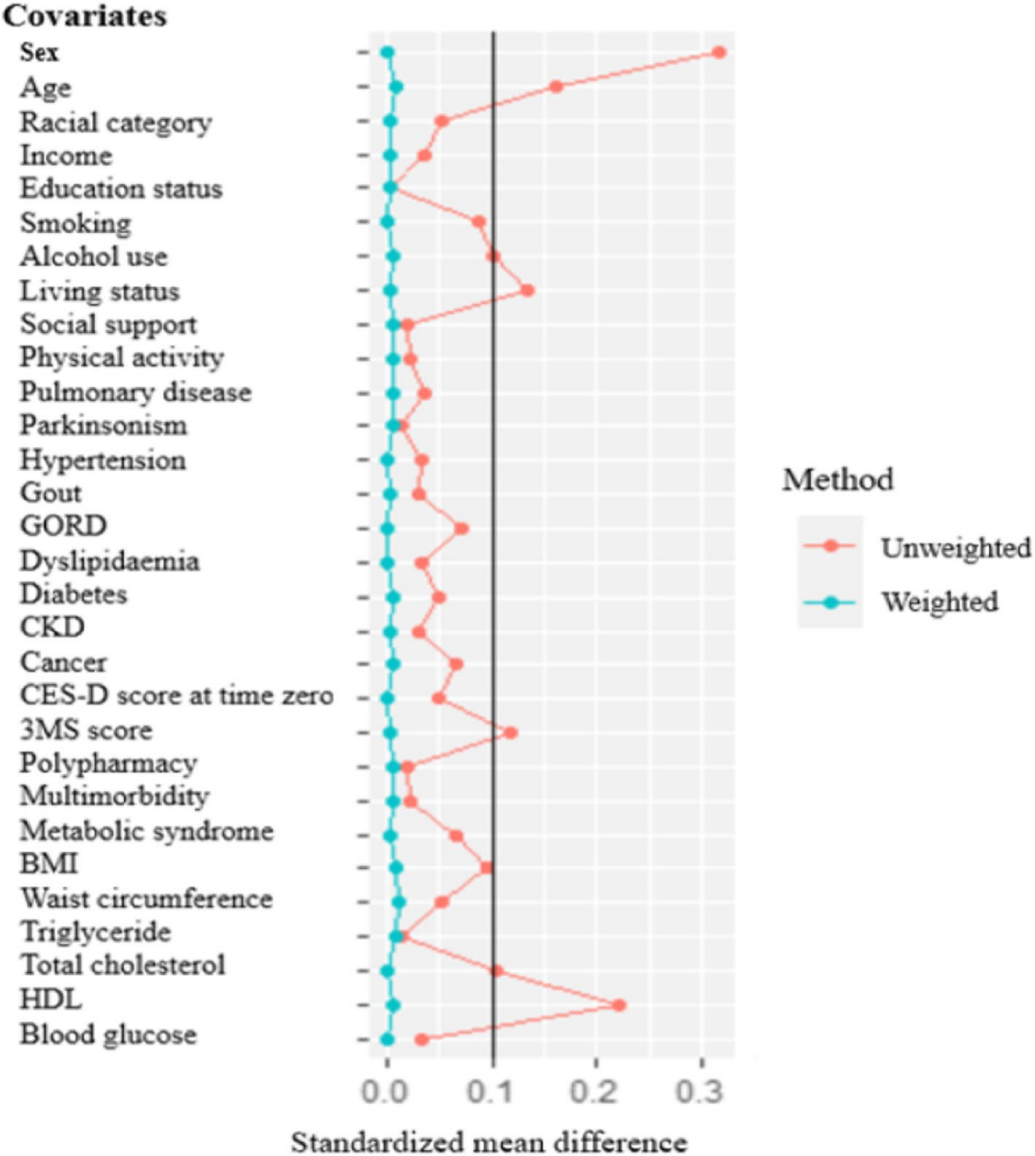
Absolute standardised mean difference (SMD) for comparisons of baseline characteristics before and after IPTW

The time zero socio-cultural, body composition characteristics, comorbidities, and medication intake of participants in the intervention and control arm were similar. However, as indicated in Table 2, participants in the control arm were more frequently females (58.5% vs 42.9%). The baseline characteristics/covariates and potential confounders were fairly similar after employing the IPTWs (Additional file 2: Fig. S2). The two groups were balanced over the intervention strategies (i.e. UPF high vs UPF low) as evidenced by a standardised mean difference of < 0.1 (Fig. 2).

### Associated effect of UPF on the risk of depressive symptoms

At time zero, 17.4% and 15.8% of participants had depressive symptoms in the intervention group and the control group, respectively. During the follow-up period (median with interquartile range: 5.8 ± 2.5 years), 4682 (41.8%) participants; 1457 (42.6%) in the intervention group and 3,225 (41.5%) in the control group had depressive symptoms over the follow-up period.

As shown in Table 3, higher UPF consumption was associated with an increased risk of depressive symptoms among older adults; the associated risk of depressive symptoms among older adults who consumed high UPF (4 or more servings per day) was increased by 10% compared to those consumed low UPF (less than 4 serving per day) (RR: 1.10; 95% CI: (1.04–1.18)). A sensitivity analysis after excluding participants taking antidepressant and/or antipsychotic medications at time zero showed the risk of depressive symptoms in the high UPF group elevated by 11% as compared to the low UPF group (RR: 1.11; 95% CI: (1.04–1.20)). Moreover, after excluding participants taking antidepressant and/or antipsychotic medications at time zero and participants with depressive symptoms at time zero, the association remained significant (RR: 1.09; 95% CI: (1.00–1.19)).

**Table 3:** Model-based risk ratios estimating the risk of depressive symptoms under UPF intervention strategies of the longitudinal cohort study in Australian older adults

Participants	Intervention strategy	Risk of depressive symptoms				
		Model 1		Model 2		
		RR (95%CI)	*p*-value	RR (95%CI)	*p*-value	*E*-value
All sample (*n* = 11,192)	Control	1 (reference)	0.003	1 (reference)	0.002	1.43
	Intervention	1.10 (1.03–1.17)		1.10 (1.04–1.18)		
Sub-group one (*n* = 9560)	Control	1 (reference)	0.013	1 (reference)	0.004	1.46
	Intervention	1.10 (1.02–1.18)		1.11 (1.04–1.20)		
Sub-group two (*n* = 8280)	Control	1 (reference)	0.099	1 (reference)	0.044	1.40
	Intervention	1.09 (0.99–1.17)		1.09 (1.00–1.19)		

Sub-group one: participants on antidepressants/antipsychotics at time zero were excluded

Sub-group two: participants on antidepressants/antipsychotics and participants with depressive symptoms (CES-D 10 ≥ 8) at time zero were excluded

In model 1, covariate age, sex, BMI, smoking, alcohol use and CES-D score at time zero were used for the inverse probability weights. Variable selection was based on the *p*-values in the propensity score model

In model 2, covariates included in model 1 plus race, living status, social support, multimorbidity, baseline cognition, metabolic syndrome, polypharmacy, income, education status, intensity of physical activity, hypertension, diabetes, cancer, CKD, GORD, dyslipidaemia, Parkinsonism, pulmonary disease, gout, waist circumference, total cholesterol, HDL, triglyceride and blood glucose level were included in the inverse probability weights

The E-value estimate for the main comparison was 1.43, in which unmeasured confounders must be correlated with both consumption of UPF and risk of depressive symptoms by a risk ratio of at least 1.43 to fully explain away the observed association indicating a substantial effect size for unmeasured confounding would be needed. This suggests that unmeasured confounding was unlikely to alter these findings.

### Sub-group analysis

We performed sub-group analyses using sex-specific UPF quartiles of serving and portion size. The associated risk of depressive symptoms among older adults in the fourth UPF serving quartile was increased by 10% as compared to those in the first quartile (RR: 1.10; 95% CI: (1.02–1.20)). Similarly, older adults in the fourth UPF portion size quartile were 1.15 times more likely to develop depressive symptoms as compared to participants in the first quartile (RR: 1.15; 95% CI: (1.04–1.26)). However, no significant relationship was observed among participants in other quartiles (q2 and q3) compared with the first quartile (Additional file 1: Table S3).

**Fig. 3:**
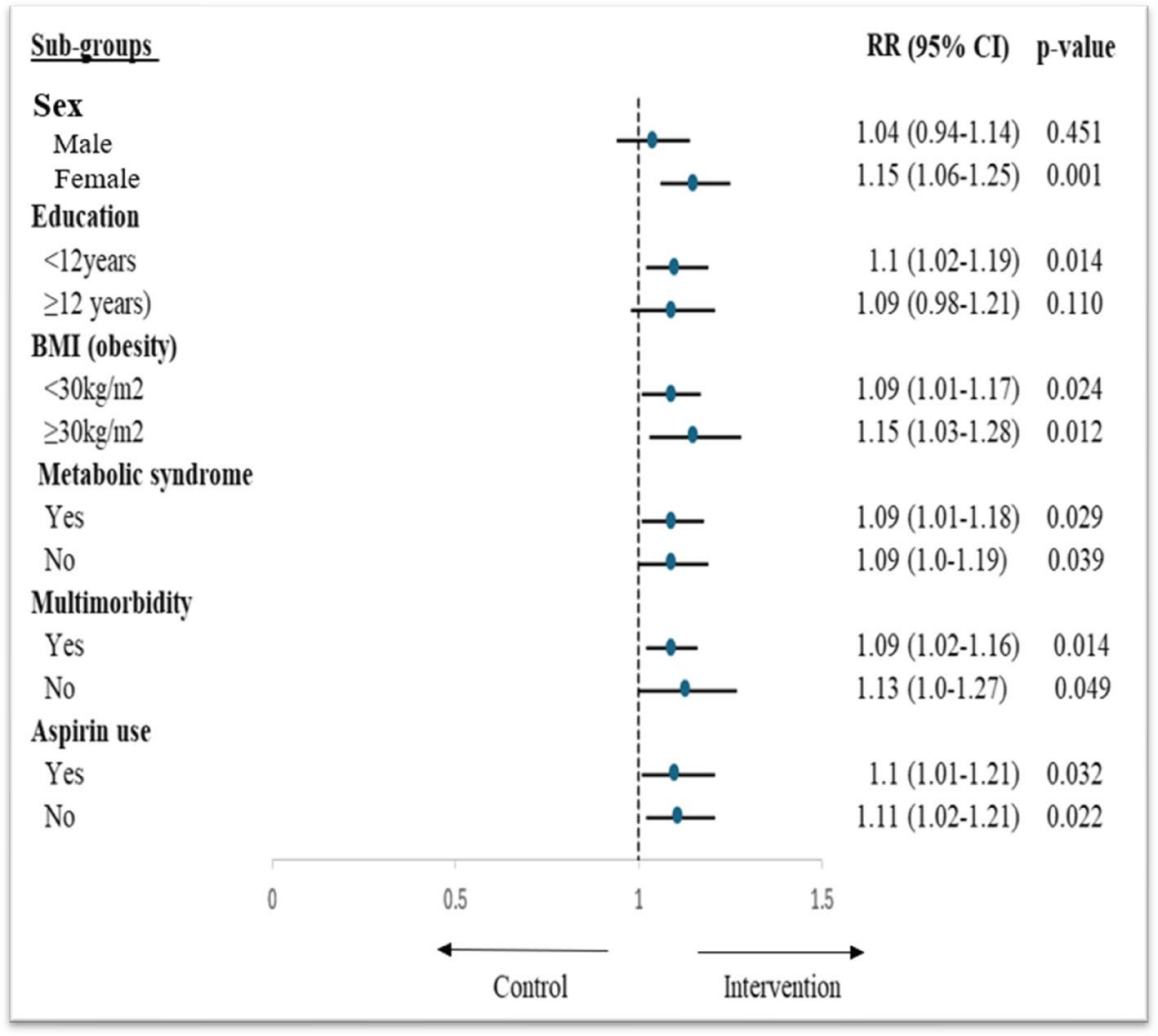
Sub-group analyses by important characteristics showing the effect of UPF consumption on risk of depressive symptoms among older adults

Sub-group analyses showed that the association between consumption of UPF and risk of depressive symptoms was significant among females and participants with lower education levels. In addition, sub-group analyses by the presence of multimorbidity and BMI categories (≥ 30 kg/m^2^ vs < 30 kg/m^2^) showed that the association was stronger among participants without multimorbidity and with higher BMI (Fig. 3).

Replicating the analyses confined to the ASPREE trial period (time zero in 2012 to 2017) indicated that high consumption of UPF adversely impacted the risk of depressive symptoms, similar to the main analysis (RR: 1.12; 95% CI: (1.05–1.20)) (Additional file 1: Table S4). Using CES-D 10 cutoff ≥ 10 (RR: 1.12; 95% CI: (1.03–1.21)) and after excluding participants on antidepressants and/or antipsychotics at time zero (RR: 1.12; 95% CI: (1.02–1.23)) consistent findings were observed with the main analysis (cutoff ≥ 8) (Additional file 1: Table S5).

### Associated effect of UPF on the SF-12 Mental Component Score

A total of 11,201 participants had a valid SF-12 score and were included in the analysis. The mean MCS was 55.6 ± SD (55.0 for intervention and 56.9 for control) at time zero and 54.6 ± SD (54.0 for intervention and 55.5 for control) at the last follow-up visit.

The between-group mean difference in MCS was significant, and there was a 0.40-unit decrease in mean MCS in the intervention group (high UPF consumption) compared to the control group (low UPF consumption) (*β*: − 0.40; 95% CI: − 0.65 to − 0.15).

Analysis using serving quartiles of UPF indicated that the between-group mean difference of MCS in the intervention group (i.e. quartile 4 of UPF serving) decreased by 0.57 units compared to the control group (*β*: − 0.57; 95% CI: − 0.89 to − 0.25). Further sub-group analysis by sex indicated that the association remains significant in females (Table 4).

**Table 4:** Model-based estimate showing the effect of UPF on mental health quality of life of Australian older adults (*n* = 11,201)

		Category	Coefficient (*β*) (95% CI)			
			Model 1	*P*-value	Model 2	*P*-value
UPF serving category (high vs low)		high vs low	− 0.63 (− 0.88 to − 0.37)	< 0.001	− 0.40 (− 0.65 to − 0.15)	0.002
UPF serving quartiles		Quartile 2 vs quartile 1	− 0.20 (− 0.51–0.10)	0.186	− 0.14 (− 0.45–0.17)	0.387
		Quartile 3 vs quartile 1	− 0.28 (− 0.58–0.03)	0.072	− 0.12 (− 0.43–0.19)	0.435
		Quartile 4 vs quartile 1	− 0.80 (− 1.11 to − 0.48)	< 0.001	− 0.57 (− 0.89 to − 0.25)	0.001
Sub-group by sex	Male	High vs low	− 0.31 (− 0.62–0.00)	0.051	− 0.17 (− 0.48–0.15)	0.304
	Female	High vs low	− 0.92 (− 1.30 to − 0.54)	< 0.001	− 0.61 (− 1.00 to − 0.23)	0.002

In model 1, covariate age, sex (not for the sub-group), BMI, smoking and alcohol use were used for the inverse probability weights

In model 2, covariates included in model 1 plus race, living status, social support, multimorbidity, baseline cognition, metabolic syndrome, MCS score at time zero, polypharmacy, income, education status, intensity of physical activity, hypertension, diabetes, cancer, CKD, GORD, dyslipidaemia, Parkinsonism, pulmonary disease, gout, waist circumference, HDL, triglyceride and blood glucose level were included in the inverse probability weights

## Discussion

In this study, we emulated a two-arm randomised pragmatic clinical trial using the ASPREE extended follow-up cohort to evaluate the effect of ultra-processed food use on the risk of depressive symptoms in older adults. We included 11,192 older adults with a median (interquartile range) follow-up of 5.8 ± 2.5 years. In the intention-to-treat analysis, a higher level of ultra-processed food consumption increased the risk of depressive symptoms and adversely affected the SF-12 mental health domain among older adults. The results were robust to challenge in multiple sensitivity analyses, and *E*-value calculation suggested the effect of unmeasured confounders is unlikely to alter the findings. Altogether, the results suggest that greater use of UPF is associated with a higher risk of depressive symptoms and poorer mental health.

Although no randomised trial has investigated the effect of UPF on depressive symptoms and mental health in older adults, prospective cohort studies on the association between UPF and depressive symptoms in the general adult population using traditional analysis have shown similar findings. The Melbourne Collaborative Cohort Study (*n* = 23,299 adults, mean age = 54.2 years) data suggested psychological distress as a marker of depression was adversely associated with UPF consumption [59]. In the Whitehall cohort study of 4554 British adults (mean age 61 years), high intake of UPF was associated with an increased likelihood of recurrent depressive symptoms over 13 years of follow-up [25]. The prospective French NutriNet_Sané study in the adult population aged 18 to 86 years (*n* = 20,380) found that an increased proportion of UPF in the diet was associated with an increased risk of depressive symptoms [60]. Although the two studies used similar depression measurement tools, the Whitehall study used the CES-D 20 ≥ 16 or antidepressant use as the endpoint, and the NutriNet_Sané used CES-D 20 ≥ 17 for males and ≥ 23 for females as cutoff points). The short format of the CES-D (CES-D 10), used in this study, has shown good predictive accuracy compared to the 20-item version [43]. Similar findings were observed in studies conducted in the Mediterranean cohort of the SUN project among 14,907 Spanish graduates (mean age = 36.7 years) [38], the Nurses’ Health Study prospective study among 31,712 middle-aged females (42–62 years) [37] and a longitudinal study of Brazilian adults (*n* = 2572, mean age = 36.1 years) [61]. All these studies were conducted among the general population (younger/ middle-aged adults).

However, a prospective cohort study using the two Brazilian birth cohorts (*n* = 3165) reported that no association was observed between quartiles of UPF consumption and incidence of common mental disorders (affective, somatic, and anhedonia symptoms) using a 20-item self-reporting questionnaire [62]. This inconsistent finding may be due to the age difference in the participants, as the latter study was conducted among a younger population (mean age = 20.8 years) in addition to the differences in outcome variables (depression vs common mental disorders). It may be the case that diet quality has a cumulative effect on health and takes several years or decades to manifest.

The main study findings were consistent with a priori sub-group analyses using various participant characteristics. Interestingly, the analysis after excluding participants who were on antidepressants and/or antipsychotics at time zero showed similar findings. We included this segment of the sample in the main analysis because there is evidence of high off-label use of antidepressants [63] and to increase the generalisability of the findings. In the analysis, after excluding participants who were on antidepressants and/or antipsychotics and those with depressive symptoms at time zero, high UPF consumption significantly increased risk of depressive symptoms, although the association was weaker.

In the sensitivity analysis using the CES-D 10 ≥ 10 cutoff score, consistent findings were observed with the main analysis (cutoff ≥ 8). Moreover, the analyses using data during the ASPREE trial period (2012–2017)) and the entire follow-up period to ASPREE-XT (2012–2022) revealed consistent findings. This analysis was conducted to ensure that diet pattern was not altered substantially over time and allows the follow-up period of this study in agreement with the follow-up periods in the previous longitudinal studies that utilised dietary data collected 4 years apart [37, 58].

Sub-group analysis by sex indicated that the association between UPF and risk of depressive symptoms was significant only among females. The non-significant finding among males may be due to insufficient statistical power as more than half of the participants (53.7%) were females. In addition, this could be due to the higher rates of depressive symptoms in females [64] which may increase the likelihood of detecting statistically significant effects in this subgroup. However, further investigation is required to verify the effect of UPF on mental health outcomes across sexes and to explore a possible underlying biological mechanism.

Analyses using quartiles of serving per day as well as portion size indicated that participants in the fourth quartile (higher servings) compared to the first quartile were at a higher risk of developing depressive symptoms over the follow-up period. This finding was in line with the findings of previous longitudinal studies [37, 65]. Although Samuthpongtorn et al.'s study was conducted only on female participants, it also highlighted that higher UPF consumption adversely affects SF-12 mental component quality of life.

Overall, the findings of this study align with existing literature confirming that high UPF consumption adversely affects mental health outcomes. The possible explanation for this relationship could be due to the fact that UPFs are high in added sugar, saturated and trans-fat, low in micronutrients (minerals and vitamins) and contain non-nutritive components such as additives (flavourings, emulsifiers, and sweeteners) [66]. This nutritional composition can lead to chronic inflammation [22, 67] which in turn may contribute to the onset of depressive symptoms [68, 69]. The opportunity cost of a diet high in ultra-processed foods may be lower consumption of nutritionally dense unprocessed foods, which likely convey health and mental health benefits. Inflammatory molecules affect neurotransmitters, neuroendocrine function, and functional brain activity, which are relevant to the physiology of mood that result in changes in emotions and behaviour including low mood, fatigue, anxiety and sleep disturbances [13, 70]. In addition, added chemicals during food processing and packaging might affect the pathways in the microbiome-gut-brain axis [22]. Moreover, dietary compounds can change antioxidant properties and promote oxidative stress (imbalance of oxidative and antioxidant processes), causing cellular injuries, which is one of the important pathways in mental health disorders including depression [13, 71, 72].

### Strengths and limitations

The unique feature of this study is that we emulated a target trial using the core design principles of the target trial methodology and applied advanced statistical techniques. To our knowledge, this is the first target trial emulation that robustly estimated the associated effect of UPF consumption on mental health outcomes and addresses the limitations of previous observational studies. A wide range of known potential confounders (socio-demographics, lifestyle, chronic medical conditions, biomarkers and medication-related factors) were collected and incorporated in balancing off the comparative groups using the IPTW approach. A relatively large sample size with a long follow-up period was another major strength.

While interpreting our findings, we need to consider the following potential limitations. First, although we used the diet screening questionnaire based on the Australian Dietary guideline, questionnaires are prone to measurement errors as a self-reported dietary assessment and risk recall bias, especially given the age of the participants. Given that the NOVA food classification system is a well-established classification system and widely used approach in research, there may still be misclassification bias in UPF labelling, and that magnitude cannot be measured. In addition, in the measurement of UPF, we could not estimate energy intake from our data; instead, we have adjusted for BMI and physical activity in the models as proxies of energy intake. In addition, we conducted a sensitivity analysis using the proportion of UPF or estimated portion size.

Second, the possibility of change in dietary habits and change in UPF use during the follow-ups was not measured. However, we conducted a sensitivity analysis over the first four years of the follow-up period, which showed consistent findings. To assess change in dietary intake over time and its impact on mental health over time, further research incorporating repeated dietary data is warranted. Third, prevalence bias, i.e. participants already adhering to a dietary pattern before study follow-up began, can introduce selection bias if the risk varies with time [73]. In our study, diet was measured at a single time point, and we cannot evaluate the effect of selection bias through a sensitivity analysis that compares those who newly switched to a diet pattern with the long-term users. So, the observed effect could be the impact of long-term habits rather than a diet-based intervention. In addition, the CES-D 10 is a validated self-reported tool to indicate the presence of clinically relevant depressive symptoms rather than a clinical diagnosis of depression.

Lastly, although we considered a comprehensive range of confounders and used the E-value to evaluate the robustness of the findings against unmeasured confounding bias, the issue of unmeasured confounding should be still considered in the interpretation of the findings. The use of trial emulation cannot resolve all potential limitations of observational studies, such as model misspecification and measurement bias.

## Conclusions

Our study provides evidence of the real-life association of UPF consumption on the risk of depressive symptoms and mental health quality of life in Australian older adults. The findings from this target trial emulation highlight the importance of reducing UPF consumption in preventing depressive symptoms and improving mental health in older adults. This provides a rationale for developing and evaluating the effectiveness of population-based and public health programmes aiming to reduce dietary UPF intake to improve the mental health of older adults. Interventional studies, such as randomised controlled dietary trials using repeated food frequency questionnaires and clinical depression diagnosis, are needed to confirm these findings and to assess the feasibility and efficacy of dietary changes in reducing depression risk in ageing populations.

## Supplementary Information

Additional file 1: Table S1-S5. Table S1. Classification of food and drink items according to the NOVA food classification system. Table S2. Characteristics of study participants according to serving quartiles of UPF. Table S3. Sensitivity analyses using UPF quartiles of serving/day and portion size as an intervention strategy. Table S4. Sensitivity analyses using ASPREE trial period. Table S5. Sensitivity analyses using CES-D 10 cutoff ≥ 10.

Additional file 2: Figures S1-S2. Figure S1. Directed acyclic graph showing the relationship of covariates with the intervention and outcome. Figure S2. Distribution of propensity score before and after adjustment.

## Data Availability

Data cannot be shared publicly for legal and ethical reasons as data are part of a large ongoing observational cohort. Data are available from Monash University for researchers who meet the criteria at https://aspree.org/aus/for-researchers/. The analysis code can be obtained from the corresponding author upon a reasonable request.

## Abbreviations

ASPREE: ASPrin in Reducing Events in the Elderly study
ALSOP: ASPREE Longitudinal Study of Older Persons
BMI: Body mass index
CES-D 10: Center for Epidemiologic Studies-Depression 10-item
CKD: Chronic kidney disease
CI: Confidence interval
GORD: Gastro-oesophageal reflux disease
IPTW: Inverse probability of treatment weighting
MSM: Marginal structural model
MCS: Mental Component Score
3MS: Modified Mini-Mental State examination
SF-12: Short Form Health Survey
SMD: Standardised mean difference
RCTs: Randomised controlled trials
RR: Risk ratio
UPF: Ultra-processed food
WHO: World Health Organisation

## References

[1] WHO. Mental health of older adults. 2023. Available at: https://www.who.int/news-room/fact-sheets/detail/mental-health-of-older-adults.

[2] Zhao Y, Wu X, Tang M, Shi L, Gong S, Mei X, et al. Late-life depression: Epidemiology, phenotype, pathogenesis and treatment before and during the COVID-19 pandemic. Front Psych. 2023;14: 1017203. 10.3389/fpsyt.2023.1017203 PMC10119596 37091719

[3] Forbes MP, O’Neil A, Lane M, Agustini B, Myles N, Berk M. Major depressive disorder in older patients as an inflammatory disorder: implications for the pharmacological management of geriatric depression. Drugs Aging. 2021;38:451–67. 33913114 10.1007/s40266-021-00858-2

[4] Xu B, Houston D, Locher JL, Zizza C. The association between Healthy Eating Index-2005 scores and disability among older Americans. Age Ageing. 2012;41(3):365–71. 22169770 10.1093/ageing/afr158

[5] Jang E-H, Han Y-J, Jang S-E, Lee S. Association between diet quality and sarcopenia in older adults: Systematic review of prospective cohort studies. Life. 2021;11(8): 811. 34440555 10.3390/life11080811 PMC8399213

[6] Davis JA, Mohebbi M, Collier F, Loughman A, Staudacher H, Shivappa N, et al. The role of diet quality and dietary patterns in predicting muscle mass and function in men over a 15-year period. Osteoporos Int. 2021;32(11):2193–203. 34043032 10.1007/s00198-021-06012-3 PMC8155648

[7] Fan Y, Zhang Y, Li J, Liu Y, Zhou L, Yu Y. Association between healthy eating index-2015 and physical frailty among the United States elderly adults: the national health and nutrition examination survey (NHANES) 2011–2014. Aging Clin Exp Res. 2021;33(12):3245–55. 33978925 10.1007/s40520-021-01874-3

[8] Lassale C, Batty GD, Baghdadli A, Jacka F, Sánchez-Villegas A, Kivimäki M, et al. Healthy dietary indices and risk of depressive outcomes: a systematic review and meta-analysis of observational studies. Mol Psychiatry. 2019;24(7):965–86. 30254236 10.1038/s41380-018-0237-8 PMC6755986

[9] Chen X, Liu Z, Sachdev P, Kochan N, O’Leary F, Brodaty H. Dietary patterns and cognitive health in older adults: findings from the Sydney Memory and Ageing Study. J Nutr Health Aging. 2021;25:255–62.33491042 10.1007/s12603-020-1536-8

[10] Chen K-H, Ho M-H, Wang C-S, Chen I-H. Effect of dietary patterns on cognitive functions of older adults: A systematic review and meta-analysis of randomized controlled trials: Dietary Patterns on Cognition of Older Adults. Arch Gerontol Geriatr. 2023:104967. 10.1016/j.archger.2023.104967 36840986

[11] Atkins JL, Whincup PH, Morris RW, Lennon LT, Papacosta O, Wannamethee SG. High diet quality is associated with a lower risk of cardiovascular disease and all-cause mortality in older men 1, 2, 3. J Nutr. 2014;144(5):673–80. 24572037 10.3945/jn.113.186486 PMC3985824

[12] Schulze MB, Martínez-González MA, Fung TT, Lichtenstein AH, Forouhi NG. Food based dietary patterns and chronic disease prevention. BMJ. 2018;361:k2396. 29898951 10.1136/bmj.k2396 PMC5996879

[13] Marx W, Lane M, Hockey M, Aslam H, Berk M, Walder K, et al. Diet and depression: exploring the biological mechanisms of action. Mol Psychiatry. 2021;26(1):134–50. 33144709 10.1038/s41380-020-00925-x

[14] Monteiro CA, Cannon G, Levy RB, Moubarac J-C, Louzada ML, Rauber F, et al. Ultra-processed foods: what they are and how to identify them. Public Health Nutr. 2019;22(5):936–41. 30744710 10.1017/S1368980018003762 PMC10260459

[15] Henney AE, Gillespie CS, Alam U, Hydes TJ, Mackay CE, Cuthbertson DJ. High intake of ultra-processed food is associated with dementia in adults: a systematic review and meta-analysis of observational studies. J Neurol. 2023:1–13. 10.1007/s00415-023-12033-1 PMC10770002 37831127

[16] Machado PP, Steele EM, Levy RB, da Costa Louzada ML, Rangan A, Woods J, et al. Ultra-processed food consumption and obesity in the Australian adult population. Nutr Diabetes. 2020;10(1):39. 33279939 10.1038/s41387-020-00141-0 PMC7719194

[17] Scrinis G, Monteiro C. From ultra-processed foods to ultra-processed dietary patterns. Nature Food. 2022;3(9):671–3. 37118150 10.1038/s43016-022-00599-4

[18] Sawalha K, Tripathi V, Alkhatib D, Alalawi L, Mahmood A, Alexander T. Our hidden enemy: Ultra-processed foods, inflammation, and the battle for heart health. Cureus. 2023;15(10):e47484. 38022349 10.7759/cureus.47484 PMC10663139

[19] Pinho MGM, Lakerveld J, Harbers MC, Sluijs I, Vermeulen R, Huss A, et al. Ultra-processed food consumption patterns among older adults in the Netherlands and the role of the food environment. Eur J Nutr. 2021;60:2567–80. 33236180 10.1007/s00394-020-02436-5 PMC8275501

[20] Mazloomi SN, Talebi S, Mehrabani S, Bagheri R, Ghavami A, Zarpoosh M, et al. The association of ultra-processed food consumption with adult mental health disorders: a systematic review and dose-response meta-analysis of 260,385 participants. Nutr Neurosci. 2022;26(10):913–31. 10.1080/1028415X.2022.2110188 36094005

[21] Elizabeth L, Machado P, Zinöcker M, Baker P, Lawrence M. Ultra-processed foods and health outcomes: a narrative review. Nutrients. 2020;12(7): 1955. 32630022 10.3390/nu12071955 PMC7399967

[22] Tristan Asensi M, Napoletano A, Sofi F, Dinu M. Low-grade inflammation and ultra-processed foods consumption: a review. Nutrients. 2023;15(6): 1546. 36986276 10.3390/nu15061546 PMC10058108

[23] Lane MM, Gamage E, Du S, Ashtree DN, McGuinness AJ, Gauci S, et al. Ultra-processed food exposure and adverse health outcomes: umbrella review of epidemiological meta-analyses. BMJ. 2024;384:e077310. 38418082 10.1136/bmj-2023-077310 PMC10899807

[24] Lane MM, Gamage E, Travica N, Dissanayaka T, Ashtree DN, Gauci S, et al. Ultra-processed food consumption and mental health: a systematic review and meta-analysis of observational studies. Nutrients. 2022;14(13): 2568. 35807749 10.3390/nu14132568 PMC9268228

[25] Arshad H, Head J, Jacka FN, Lane MM, Kivimaki M, Akbaraly T. Association between ultra-processed foods and recurrence of depressive symptoms: the Whitehall II cohort study. Nutritional Neuroscience. 2023:1–13. 10.1080/1028415X.2022.2157927 PMC10679838 36989349

[26] Bhattarai N, Prevost AT, Wright AJ, Charlton J, Rudisill C, Gulliford MC. Effectiveness of interventions to promote healthy diet in primary care: systematic review and meta-analysis of randomised controlled trials. BMC Public Health. 2013;13:1–14. 24355095 10.1186/1471-2458-13-1203 PMC3890643

[27] Satija A, Stampfer MJ, Rimm EB, Willett W, Hu FB. Perspective: are large, simple trials the solution for nutrition research? Adv Nutr. 2018;9(4):378–87. 30032229 10.1093/advances/nmy030 PMC6054238

[28] Mirmiran P, Bahadoran Z, Gaeini Z. Common limitations and challenges of dietary clinical trials for translation into clinical practices. Int J Endocrinol Metab. 2021;19(3):e108170. 34567133 10.5812/ijem.108170 PMC8453651

[29] Hernan MA, Robins JM. Causal inference: What if. Boca Raton: Chapman & Hill/CRC; 2020.

[30] Chesnaye NC, Stel VS, Tripepi G, Dekker FW, Fu EL, Zoccali C, et al. An introduction to inverse probability of treatment weighting in observational research. Clin Kidney J. 2022;15(1):14–20. 35035932 10.1093/ckj/sfab158 PMC8757413

[31] ASPREE Investigator Group. Study design of ASPirin in Reducing Events in the Elderly (ASPREE): a randomized, controlled trial. Contemp Clin Trials. 2013;36(2):555–64. 24113028 10.1016/j.cct.2013.09.014 PMC3919683

[32] McNeil JJ, Woods RL, Nelson MR, Murray AM, Reid CM, Kirpach B, et al. Baseline characteristics of participants in the ASPREE (ASPirin in Reducing Events in the Elderly) study. The Journals of Gerontology: Series A. 2017;72(11):1586–93. 10.1093/gerona/glw342 PMC5861878 28329340

[33] Berk M, Woods RL, Nelson MR, Shah RC, Reid CM, Storey E, et al. ASPREE-D: Aspirin for the prevention of depression in the elderly. Int Psychogeriatr. 2016;28(10):1741–8. 27587328 10.1017/S104161021600079X PMC6719794

[34] McNeil JJ, Woods RL, Ward SA, Britt CJ, Lockery JE, Beilin LJ, et al. Cohort Profile: The ASPREE Longitudinal Study of Older Persons (ALSOP). Int J Epidemiol. 2019;48(4):1048–9. 30624660 10.1093/ije/dyy279 PMC6693806

[35] Ernst ME, Broder JC, Wolfe R, Woods RL, Nelson MR, Ryan J, et al. Health characteristics and aspirin use in participants at the baseline of the aspirin in reducing events in the elderly–eXTension (ASPREE-XT) observational study. Contemp Clin Trials. 2023;130: 107231. 37196887 10.1016/j.cct.2023.107231 PMC10330669

[36] Cancer Council Victoria. Dietary questionnaire for epidemiological studies version 3.2 (DQES v3.2) user guide. 2023.

[37] Samuthpongtorn C, Nguyen LH, Okereke OI, Wang DD, Song M, Chan AT, et al. Consumption of ultraprocessed food and risk of depression. JAMA Network Open. 2023;6(9):e2334770-e. 37728928 10.1001/jamanetworkopen.2023.34770 PMC10512104

[38] Gómez-Donoso C, Sánchez-Villegas A, Martínez-González MA, Gea A, Mendonça RdD, Lahortiga-Ramos F, et al. Ultra-processed food consumption and the incidence of depression in a Mediterranean cohort: the SUN Project. European journal of nutrition. 2020;59:1093–103. 31055621 10.1007/s00394-019-01970-1

[39] Zheng M, Wu JH, Louie JCY, Flood VM, Gill T, Thomas B, et al. Typical food portion sizes consumed by Australian adults: results from the 2011–12 Australian National Nutrition and Physical Activity Survey. Sci Rep. 2016;6(1):19596. 26786684 10.1038/srep19596 PMC4726402

[40] Li H, Li S, Yang H, Zhang Y, Zhang S, Ma Y, et al. Association of ultraprocessed food consumption with risk of dementia: a prospective cohort study. Neurology. 2022;99(10):e1056–66. 36219796 10.1212/WNL.0000000000200871

[41] Contreras-Rodriguez O, Reales-Moreno M, Fernández-Barrès S, Cimpean A, Arnoriaga-Rodríguez M, Puig J, et al. Consumption of ultra-processed foods is associated with depression, mesocorticolimbic volume, and inflammation. J Affect Disord. 2023;335:340–8. 37207947 10.1016/j.jad.2023.05.009

[42] Wang X, Wu Y, Miao J, Pu K, Ming W-K, Zang S. Factors associated with eating behaviors in older adults from a socioecological model perspective. BMC Public Health. 2023;23(1):1726. 37670266 10.1186/s12889-023-16651-2 PMC10481492

[43] Andresen EM, Malmgren JA, Carter WB, Patrick DL. Screening for depression in well older adults: evaluation of a short form of the CES-D (Center for Epidemiologic Studies Depression Scale). Am J Prev Med. 1994;10(2):77–84. 8037935

[44] Mohebbi M, Nguyen V, McNeil JJ, Woods RL, Nelson MR, Shah RC, et al. Psychometric properties of a short form of the Center for Epidemiologic Studies Depression (CES-D-10) scale for screening depressive symptoms in healthy community dwelling older adults. Gen Hosp Psychiatry. 2018;51:118–25. 28890280 10.1016/j.genhosppsych.2017.08.002 PMC6178798

[45] Mohebbi M, Agustini B, Woods RL, McNeil JJ, Nelson MR, Shah RC, et al. Prevalence of depressive symptoms and its associated factors among healthy community-dwelling older adults living in Australia and the United States. Int J Geriatr Psychiatry. 2019;34(8):1208–16. 30989707 10.1002/gps.5119 PMC6924573

[46] Agustini B, Mohebbi M, Woods RL, McNeil JJ, Nelson MR, Shah RC, et al. The association of antihypertensive use and depressive symptoms in a large older population with hypertension living in Australia and the United States: a cross-sectional study. J Hum Hypertens. 2020;34(11):787–94. 32001828 10.1038/s41371-020-0303-y PMC7390661

[47] Sanderson K, Andrews G. The SF-12 in the Australian population: cross-validation of item selection. Aust N Z J Public Health. 2002;26(4):343–5. 12233955 10.1111/j.1467-842x.2002.tb00182.x

[48] Gandek B, Ware JE, Aaronson NK, Apolone G, Bjorner JB, Brazier JE, et al. Cross-validation of item selection and scoring for the SF-12 Health Survey in nine countries: results from the IQOLA Project. J Clin Epidemiol. 1998;51(11):1171–8. 9817135 10.1016/s0895-4356(98)00109-7

[49] Vilagut G, Forero CG, Pinto-Meza A, Haro JM, De Graaf R, Bruffaerts R, et al. The mental component of the short-form 12 health survey (SF-12) as a measure of depressive disorders in the general population: results with three alternative scoring methods. Value in Health. 2013;16(4):564–73. 23796290 10.1016/j.jval.2013.01.006

[50] Lubben J, Blozik E, Gillmann G, Iliffe S, von Renteln KW, Beck JC, et al. Performance of an abbreviated version of the Lubben Social Network Scale among three European community-dwelling older adult populations. Gerontologist. 2006;46(4):503–13. 16921004 10.1093/geront/46.4.503

[51] Huang PL. A comprehensive definition for metabolic syndrome. Dis Model Mech. 2009;2(5–6):231–7. 19407331 10.1242/dmm.001180 PMC2675814

[52] Stürmer T, Wyss R, Glynn RJ, Brookhart MA. Propensity scores for confounder adjustment when assessing the effects of medical interventions using nonexperimental study designs. J Intern Med. 2014;275(6):570–80. 24520806 10.1111/joim.12197 PMC4037382

[53] Pezzi A, Cavo M, Biggeri A, Zamagni E, Nanni O. Inverse probability weighting to estimate causal effect of a singular phase in a multiphase randomized clinical trial for multiple myeloma. BMC Med Res Methodol. 2016;16:1–10. 27829371 10.1186/s12874-016-0253-9 PMC5103416

[54] Klungsøyr O, Fredriksen M. Pharmacological Treatment of Adult Attention-Deficit/Hyperactivity Disorder (ADHD) in a Longitudinal Observational Study: Estimated Treatment Effect Strengthened by Improved Covariate Balance. Open J Stat. 2017;7(6):988–1012.

[55] Austin PC, Stuart EA. Moving towards best practice when using inverse probability of treatment weighting (IPTW) using the propensity score to estimate causal treatment effects in observational studies. Stat Med. 2015;34(28):3661–79. 26238958 10.1002/sim.6607 PMC4626409

[56] Mathur MB, Ding P, Riddell CA, VanderWeele TJ. Web Site and R Package for Computing E-values. Epidemiology. 2018;29(5):e45–7. 29912013 10.1097/EDE.0000000000000864 PMC6066405

[57] VanderWeele TJ, Ding P. Sensitivity analysis in observational research: introducing the E-value. Ann Intern Med. 2017;167(4):268–74. 28693043 10.7326/M16-2607

[58] Gonçalves NG, Ferreira NV, Khandpur N, Steele EM, Levy RB, Lotufo PA, et al. Association between consumption of ultraprocessed foods and cognitive decline. JAMA Neurol. 2023;80(2):142–50. 36469335 10.1001/jamaneurol.2022.4397 PMC9857155

[59] Lane MM, Lotfaliany M, Hodge AM, O’Neil A, Travica N, Jacka FN, et al. High ultra-processed food consumption is associated with elevated psychological distress as an indicator of depression in adults from the Melbourne Collaborative Cohort Study. J Affect Disord. 2023;335:57–66. 37149054 10.1016/j.jad.2023.04.124

[60] Adjibade M, Julia C, Allès B, Touvier M, Lemogne C, Srour B, et al. Prospective association between ultra-processed food consumption and incident depressive symptoms in the French NutriNet-Santé cohort. BMC Med. 2019;17:1–13. 30982472 10.1186/s12916-019-1312-y PMC6463641

[61] Leal ACG, Lopes LJ, Rezende-Alves K, Bressan J, Pimenta AM, Hermsdorff HHM. Ultra-processed food consumption is positively associated with the incidence of depression in Brazilian adults (CUME project). J Affect Disord. 2023;328:58–63. 36791971 10.1016/j.jad.2023.01.120

[62] Werneck AO, Costa CS, Horta B, Wehrmeister FC, Gonçalves H, Menezes AMB, et al. Prospective association between ultra-processed food consumption and incidence of elevated symptoms of common mental disorders. J Affect Disord. 2022;312:78–85. 35691417 10.1016/j.jad.2022.06.007

[63] Skånland SS, Cieślar-Pobuda A. Off-label uses of drugs for depression. Eur J Pharmacol. 2019;865: 172732. 31622593 10.1016/j.ejphar.2019.172732

[64] Mengist B, Lotfaliany M, Pasco JA, Agustini B, Berk M, Williams LJ, et al. Gait speed, handgrip strength, and their combination, and risk of depression in later life: Evidence from a prospective study of community-dwelling older adults. J Affect Disord. 2024;369:218–26. 39353510 10.1016/j.jad.2024.09.155

[65] Zheng L, Sun J, Yu X, Zhang D. Ultra-processed food is positively associated with depressive symptoms among United States adults. Front Nutr. 2020;7: 600449. 33385006 10.3389/fnut.2020.600449 PMC7770142

[66] Martini D, Godos J, Bonaccio M, Vitaglione P, Grosso G. Ultra-processed foods and nutritional dietary profile: a meta-analysis of nationally representative samples. Nutrients. 2021;13(10): 3390. 34684391 10.3390/nu13103390 PMC8538030

[67] Quetglas-Llabrés MM, Monserrat-Mesquida M, Bouzas C, Mateos D, Ugarriza L, Gómez C, et al. Oxidative stress and inflammatory biomarkers are related to high intake of ultra-processed food in old adults with metabolic syndrome. Antioxidants. 2023;12(8): 1532. 37627527 10.3390/antiox12081532 PMC10451674

[68] Tiemeier H, Hofman A, van Tuijl HR, Kiliaan AJ, Meijer J, Breteler MM. Inflammatory proteins and depression in the elderly. Epidemiology. 2003;14(1):103–7. 12500057 10.1097/00001648-200301000-00025

[69] Bondy E, Norton SA, Voss M, Marks RB, Boudreaux MJ, Treadway MT, et al. Inflammation is associated with future depressive symptoms among older adults. Brain, Behavior, & Immunity-Health. 2021;13: 100226. 34589741 10.1016/j.bbih.2021.100226 PMC8474183

[70] Köhler-Forsberg O N, Lydholm C, Hjorthøj C, Nordentoft M, Mors O, Benros M. Efficacy of anti-inflammatory treatment on major depressive disorder or depressive symptoms: meta-analysis of clinical trials. Acta Psychiatrica Scandinavica. 2019;139(5):404–19. 30834514 10.1111/acps.13016

[71] Moylan S, Berk M, Dean OM, Samuni Y, Williams LJ, O’Neil A, et al. Oxidative & nitrosative stress in depression: why so much stress? Neurosci Biobehav Rev. 2014;45:46–62. 24858007 10.1016/j.neubiorev.2014.05.007

[72] Studzinski CM, Li F, Bruce-Keller AJ, Fernandez-Kim SO, Zhang L, Weidner AM, et al. Effects of short-term Western diet on cerebral oxidative stress and diabetes related factors in APP× PS1 knock-in mice. J Neurochem. 2009;108(4):860–6. 19046405 10.1111/j.1471-4159.2008.05798.x PMC2748316

[73] Hernán MA, Hernández-Díaz S, Robins JM. A structural approach to selection bias. Epidemiology. 2004;15(5):615–25. 15308962 10.1097/01.ede.0000135174.63482.43

