# Supplementary material for: The risk associated with ultra-processed food intake on depressive symptoms and mental health in older adults: a target trial emulation
Source: BMC Med. 2025 Mar 24;23:172. doi: 10.1186/s12916-025-04002-4 (PMC11934811; doi:10.1186/s12916-025-04002-4)
Supplement: Supplementary file 1 — Additional file 1: Table S1-S5. Table S1. Classification of food and drink items according to the NOVA food classification system. Table S2. Characteristics of study participants according to serving quartiles of UPF. Table S3. Sensitivity analyses using UPF quartiles of serving/day and portion size as an intervention strategy. Table S4. Sensitivity analyses using ASPREE trial period. Table S5. Sensitivity analyses using CES-D 10 cutoff ≥ 10. [file 12916_2025_4002_MOESM1_ESM.docx]

**Additional file 1**

**Table S1:** Classification of food and drink items according to the NOVA food classification system.

| **NOVA group** | **Food group** | **Food and drink items** |
| --- | --- | --- |
| Group 1 | Unprocessed or minimally processed foods, | Rice, pasta / noodles, fresh fruit, raw/salad vegetables, cooked green vegetables, cooked orange/ yellow / red vegetables (e.g. carrots, beets), potatoes, beans, and legumes (e.g. peas, lentils), yoghurt,  Red meat (not corned, pickled, or processed), Chicken, turkey or other poultry, eggs, Deep fried/battered fish, Salmon, mackerel or other oily fish (not including deep fried fish), White fish (not including deep fried fish),  Other seafood (e.g. prawns or shellfish), nuts, fruit juice, low-fat or skim milk, full-cream milk, water, coffee, tea, herbal tea |
| Group 2 | Processed culinary ingredients | Olive oil, butter, other vegetable oil, salt |
| Group 3 | Processed foods | Cheese, cream, cream cheese or similar, cooked, tinned or dried fruit, tinned fish (e.g. canned salmon, tuna, sardines). |
| Group 4 | Ultra-processed food | Soy or other non-dairy milk, malt drinks (e.g. Milo or Horlicks), hot chocolate, cordial, soft drink (e.g. regular Coke), diet Soft drink (e.g. diet Coke), Supplement drink (e.g. Ensure or Sustagen), sausages, processed meats (e.g. bacon, ham, corned beef or salami), potato chips or similar, sweet biscuits/ cakes, dark chocolate, milk chocolate, lollies or other sweets, hamburgers, pizza or 'fast' food, meat pies (sausage rolls etc), ice cream, frozen yoghurt or other dairy desserts, white bread, brown/multigrain bread, breakfast cereal/ oats, crackers / savoury biscuits. |

**Table S2**: Characteristics of study participants according to serving quartiles of UPF consumption.

| **Covariates** | | **Overall cohort** (frequency/%) | **UPF consumption (quartiles)** | | | |
| --- | --- | --- | --- | --- | --- | --- |
|  |  |  | Q1 (frequency, %) | Q2 (frequency, %) | Q3 (frequency, %) | Q4 (frequency, %) |
| Sex | Male | 5,175 (46.3) | 1,300 (25.12) | 1,288 (24.89) | 1,294 (25.0) | 1,293 (24.99) |
|  | Female | 6,017 (53.7) | 1,506/25.03 | 1,503/24.98 | 1,504 (25.0) | 1,504 (25.0) |
| Education status | ≤12years | 6,509 (58.2) | 1,628 (25.01) | 1,627 (25.0) | 1,627 (25.0) | 1,627 (25.0) |
|  | >12years | 4,682 (41.8) | 1,171 (25.01) | 1,170 (25.0) | 1,171 (25.01) | 1,170 (25.0) |
| Racial | White | 11,059/98.8 | 2,767 (25.02) | 2,764 (24.99) | 2,764 (24.99) | 2,764 (24.99) |
|  | Non-white | 130 (1.2) | 33 (25.38) | 32 (24.62) | 33 (25.38) | 32 (24.62) |
| Alcohol | Yes | 8,322 (74.8) | 2,081 (25.01) | 2,080 (24.99) | 2,081 (25.01) | 2,080 (24.99) |
|  | No | 2,807 (25.2) | 702 (25.01) | 702 (25.01) | 702 (25.01) | 701 (24.97) |
| Smoking | Yes | 252/2.3 | 63 (25.0) | 63 (25.0) | 63 (25.0) | 63 (25.0) |
|  | No | 10,877 (97.7) | 2,720 (25.01) | 2,721 (25.02) | 2,725 (25.05) | 2,711 (24.92) |
| Income | Inadequate | 688 (7.2) | 172 (25.0) | 172 (25.0) | 173 (25.15) | 171 (24.85) |
|  | Adequate | 8,855 (92.8) | 2,217 (25.04) | 2,212 (24.98) | 2,215 (25.01) | 2,211 (24.97) |
| Social support | Poor | 938 (9.9) | 238 (25.37) | 231 (24.63) | 235 (25.05) | 234 (24.95) |
|  | Good | 8,490/90.1 | 2,123 (25.01) | 2,123 (25.01) | 2,125 (25.03) | 2,119 (24.96) |
| Living status | at home with family | 7,525/67.6 | 1,882 (25.01) | 1,883 (25.02) | 1,879 (24.97) | 1,881 (25.0) |
|  | At home alone/ residential | 3,607 (32.4) | 902 (25.01) | 905 (25.09) | 902 (25.01) | 898 (24.9) |
| Physical activity | Low | 3,673 (38.2) | 929 (25.29) | 910 (24.78) | 918 (24.99) | 916 (24.94) |
|  | Moderate to high | 5,953 (61.8) | 1,489 (25.01) | 1,492 (25.06) | 1,488 (25.0) | 1,484 (24.93) |
| Metabolic syndrome | Yes | 5,000 (54.8) | 1,250 (25.0) | 1,253 (25.06) | 1,247 (24.94) | 1,250 (25.0) |
|  | No | 4,131 (45.2) | 1,033 (25.01) | 1,033 (25.01) | 1,033 (25.01) | 1,032 (24.98) |
| Multimorbidity | Yes | 7,523 (81.5) | 1,882 (25.02) | 1,881 (25.0) | 1,880 (24.99) | 1,880 (24.99) |
|  | No | 1,704 (18.5) | 426 (25.0) | 426 (25.0) | 426 (25.0) | 426 (25.0) |
| Polypharmacy | Yes | 4,665 (41.7) | 1,168 (25.04) | 1,165 (24.97) | 1,166 (24.99) | 1,166 (24.99) |
|  | No | 6,519 (58.3) | 1,632 (25.03) | 1,630 (25.00) | 1,628 (24.97) | 1,629 (24.99) |

**Table S3:** Sensitivity analyses using UPF quartiles of serving/day and portion size as an intervention strategy.

| **Participants** | **Quartiles** | **Serving/day** | | **Portion size** | |
| --- | --- | --- | --- | --- | --- |
|  |  | RR (95%CI) | p-value | RR (95%CI) | p-value |
| All sample | Q1 | 1.00 | Reference | 1.00 | Reference |
|  | Q2 | 1.01(0.93-1.09) | 0.901 | 1.00(0.91-1.10) | 0.967 |
|  | Q3 | 0.97(0.89-1.05) | 0.408 | 1.03(0.94-1.13) | 0.533 |
|  | Q4 | 1.10(1.02-1.20) | 0.020 | 1.15(1.04-1.26) | 0.006 |
| Sub-group one | Q1 | 1.00 | Reference | 1.00 | Reference |
|  | Q2 | 1.02(.93-1.13) | 0.619 | 0.97(.89-1.09) | 0.632 |
|  | Q3 | 1.01(0.92-1.11) | 0.840 | 1.04(0.93-1.16) | 0.499 |
|  | Q4 | 1.11(1.01-1.22) | 0.032 | 1.12(1.00-1.26) | 0.052 |
| Sub-group two | Q1 | 1.00 | Reference | 1.00 | Reference |
|  | Q2 | 1.02(0.92-1.15) | 0.667 | 0.99(0.88-1.13) | 0.937 |
|  | Q3 | 1.00(0.90-1.12) | 0.933 | 1.06(0.93-1.20) | 0.400 |
|  | Q4 | 1.10(0.98-1.24) | 0.093 | 1.16(1.01-1.32) | 0.039 |

- IPTW for age, sex, BMI, smoking, alcohol use, race, living status, social support, multimorbidity, baseline cognition, CES-D score at time zero, metabolic syndrome, polypharmacy, income, education status, intensity of physical activity, hypertension, diabetes, cancer, CKD, GORD, dyslipidaemia, Parkinsonism, pulmonary disease, gout, waist circumference, total cholesterol, HDL, triglyceride and blood glucose level.
- **Sub-group** **one:** participants on antidepressants/antipsychotics at time zero were excluded.
- **Sub-group two:** participants on antidepressants/antipsychotics and participants with depressive symptoms (CES-D 10 ≥8) at time zero were excluded.

**Table S4**: Sensitivity analyses using ASPREE trial period (from 2012 to 2017)

| **Participants** | **Intervention strategy** | **Risk of depressive symptoms** | |
| --- | --- | --- | --- |
|  |  | RR (95%CI) | p-value |
| All sample | Control | 1 (reference) | 0.001 |
|  | Intervention | 1.12 (1.05-1.20) |  |
| Sub-group one | Control | 1 (reference) | 0.002 |
|  | Intervention | 1.13 (1.05- 1.23) |  |
| Sub-group two | Control | 1 (reference) | 0.062 |
|  | Intervention | 1.10 (1.00- 1.21) |  |

- Age, sex, BMI, smoking, alcohol use, race, living status, social support, multimorbidity, baseline cognition, CES-D score at time zero, metabolic syndrome, polypharmacy, income, education status, intensity of physical activity, hypertension, diabetes, cancer, CKD, GORD, dyslipidaemia, Parkinsonism, pulmonary disease, gout, waist circumference, total cholesterol, HDL, triglyceride and blood glucose level were included in the inverse probability weights.
- **Sub-group** **one:** participants on antidepressants/antipsychotics at time zero were excluded.
- **Sub-group** **two:** participants on antidepressants/antipsychotics and participants with depressive symptoms (CES-D 10 ≥8) at time zero were excluded.

**Table S5**: Sensitivity analyses using CES-D 10 cutoff $\geq10$

| **Participants** | **Intervention strategy** | **Risk of depressive symptoms** | |
| --- | --- | --- | --- |
| All sample |  | RR (95%CI) | p-value |
|  | Control | 1 (reference) | 0.007 |
|  | Intervention | 1.12 (1.03-1.21) |  |
| Sub-group one | Control | 1 (reference) | 0.020 |
|  | Intervention | 1.12 (1.02- 1.23) |  |
| Sub-group two | Control | 1 (reference) | 0.168 |
|  | Intervention | 1.08 (0.97- 1.20) |  |

- Age, sex, BMI, smoking, alcohol use, race, living status, social support, multimorbidity, baseline cognition, CES-D score at time zero, metabolic syndrome, polypharmacy, income, education status, intensity of physical activity, hypertension, diabetes, cancer, CKD, GORD, dyslipidaemia, Parkinsonism, pulmonary disease, gout, waist circumference, total cholesterol, HDL, triglyceride and blood glucose level were included in the inverse probability weights.
- **Sub-group** **one:** participants on antidepressants/antipsychotics at time zero were excluded.
- **Sub-group** **two:** participants on antidepressants/antipsychotics and participants with depressive symptoms (CES-D 10 ≥10) at time zero were excluded.
