# Supplementary material for: The risk associated with ultra-processed food intake on depressive symptoms and mental health in older adults: a target trial emulation
Source: BMC Med. 2025 Mar 24;23:172. doi: 10.1186/s12916-025-04002-4 (PMC11934811; doi:10.1186/s12916-025-04002-4)
Supplement: Supplementary file 2 — Additional file 2: Figures S1-S2. Figure S1. Directed acyclic graph showing the relationship of covariates with the intervention and outcome. Figure S2. Distribution of propensity score before and after adjustment. [file 12916_2025_4002_MOESM2_ESM.docx]

**Additional file 2**


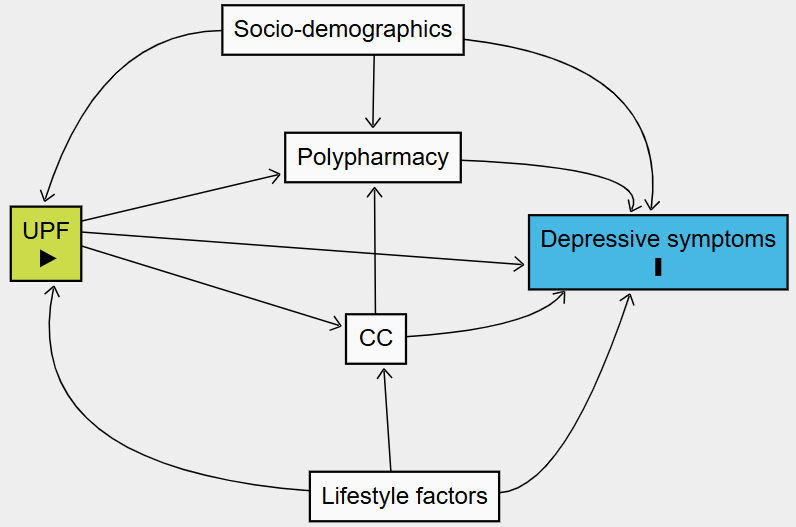


CC: Chronic conditions; including multimorbidity and metabolic syndrome, UPF: ultra-processed food

**Figure S1**: Directed acyclic graph (DAG) showing the relationship of covariates with the intervention (UPF) and outcome (depressive symptoms)


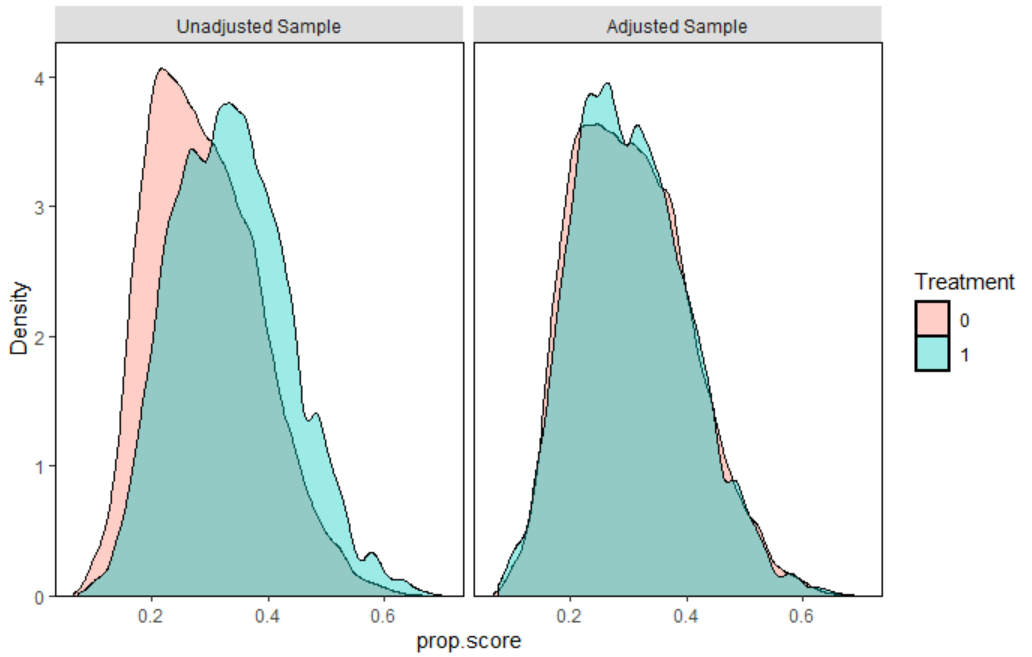


**Figure S2**: Distribution of propensity score before and after adjustment
